# Exploring the Road of Women to Medical Leadership: A European Perspective

**DOI:** 10.2196/49247

**Published:** 2023-07-05

**Authors:** Ieva Ruza

**Affiliations:** 1 Riga East Clinical University Hospital Riga Latvia

**Keywords:** women, leadership, gender pay gap, childcare, mentorship, medicine, European perspective

## Abstract

This paper addresses and discusses several issues and perspectives for women in medical leadership from the European perspective.

## Introduction

You had to think of contraception if you wanted to write your Ph.D. in Germany

Germany, 1998

Twenty-five years ago, after informing my professor about my pregnancy, those words clearly depicted my chances to fulfill my dream to perform high-quality scientific lab research, defend my thesis, and return home to become the youngest medical professor in Latvia.

With the EU labor market not yet widely opened, after my graduation as a medical doctor, I joined my husband who was close to completing his postdoctoral in physics in Germany. Learning about my pregnancy gave me a new motivation to complete the lab work faster as our family goal was to return to Latvia. However, due to various administrative barriers in Germany, I ended up signing a nondisclosure agreement and postponing my research plans. Sometimes I admit in public that my oldest child is my thesis.

Many female colleagues could add their stories of being depreciated, excluded from professional networking, or denied promotions. They might tell about being banned from projects and educational opportunities due to their current or assumed family situation.

Over the years, much has improved. More flexibility, support, and opportunities have been given for the career paths of young women in medicine at all stages; however, there are many issues to address.

## Sacrifices of Women in Medical Leadership

Trying to climb the career ladder in medicine and simultaneously achieve a balance between family and work, some women might encounter various other losses. They sacrifice their relationships with families or friends or might be confronted with far more difficult decisions about their offspring. There are still ongoing heated debates about the issue of abortion not only in many US states but also in some European countries (Poland, Ireland) [1], where women’s right to make decisions on their sexual and reproductive health should be respected.

Some studies report up to a 32% higher risk of miscarriage in women who had worked two or more night shifts the previous week when compared with women who had no night shifts that week [2].

Women might be restricted from pursuing their initial professional dreams and goals regardless of their relationship status. However, thinking about medical or academic career paths, family can play a role in decision-making. It might hinder engagement in scientific or clinical research, or professional development as decisions frequently must involve family members. Occasionally, international working or scholarship opportunities can be missed.

## Childcare Stress

In a recent *Journal of the American Medical Association* (*JAMA*) study, it was reported that approximately 21% of all health care workers recently experienced childcare stress. They had 80% greater odds of burnout and 115% greater odds of anxiety and depression, statistically more so in women than in men. Women were also more likely to report intentions to reduce work hours [3].

While maternity leave is a massive problem in the United States, many European countries have implemented flexible regulations and schemes for parental leaves [4].

However, as reported in a recent Organisation for Economic Co-operation and Development (OECD) data set, net childcare costs are soaring in Europe where Cyprus, the Czech Republic, the United Kingdom, and Ireland have the most expensive childcare in the world behind the United States and New Zealand [5]. Some strategies to address these risks and help female health care workers could be by providing flexible on-site childcare opportunities, subsidizing some alternative options, or offering some tax discounts.

## Career Opportunities in the Context of Hierarchy and Geopolitical Issues

At the European level, we frequently observe a huge diversity between the cultural traditions of geopolitical regions.

In European countries with stronger hierarchies, one can more frequently observe men being promoted to leadership positions in clinical, academic, and administrative work. Despite loud slogans and nicely formulated strategic road maps, women need to overcome various biases.

The findings of one study from 2014 suggested that, in spite of a high proportion of women in the physician workforce, they are underrepresented in leadership positions [6] or have decreased project lead opportunities.

A large study published in *The New England Journal of Medicine* (*NEJM*) in 2020 reported that over a 35-year period, women physicians in academic medical centers in the United States were less likely than men to be promoted to the rank of associate or full professor, or to be appointed to the department chair, and there was no apparent narrowing in the gap over time [7].

Not only the underrepresentation of women in various leadership positions but also massive gender pay disparities are observed throughout various sectors. While the mean gender pay gap in the European Union in 2021 is reported to be around 13%, it reaches 18% and 22% in Germany and Latvia, respectively [8]. About one-quarter of the gap is related to the overrepresentation of women in relatively low-paying industries, including health care and education [8].

## Scientific Degree

While clinical and academic career pathways seem to be rather separated in the United States, a doctoral degree (PhD) is frequently required in European countries to proceed with a professional medical career even in nonscientific administrative posts or ones only remotely connected with ongoing research.

Frequently, academic careers in medicine are never continued after being awarded a PhD degree and getting promoted to an administrative post. Occasionally, maintaining academic status might lead to various foul practices, like adding surnames to publications without actual involvement in research. Local regulations and cultural traditions in some European countries can be so tight that they prevent transparent and innovative leadership workforce mobility and add additional pressure for women on the road to medical leadership positions.

There is a necessity to cultivate transgenerational transfer of knowledge and expertise, developing professional networks.

## Allyship, Mentorship, and Sponsorship

There are no officially established traditions of mentoring and sponsoring in most European countries, and the way these relationships are developing differs regionally.

While many of us have carved our initial way upward without the help of other people, at some higher career stages, corporate culture, traditions of mentoring and sponsoring, as well as additional network opportunities play an increasingly important role.

Medical professional societies or federations need to encourage and facilitate the development of such relationships not only in moments of crisis. More support at various levels should be given for women to successfully proceed to medical clinical and academic leadership positions.

## Perspectives to Support Women’s Leadership

The following 5 perspectives can support women’s leadership:

1. Encourage an inclusive workplace culture at all leadership levels
2. Educational and professional development opportunities
3. Childcare support at various levels
4. Address the gender pay gap
5. Create and encourage more transparent allyship, mentorship, and sponsorship opportunities in the European medical leadership at all levels, beyond specific specialty fields and health care sectors

## Abbreviations

JAMA: Journal of the American Medical Association
NEJM: New England Journal of Medicine
OECD: Organisation for Economic Co-operation and Development
